# Supplementary material for: Complexome profiling of the Chlamydomonas psb28 mutant reveals TEF5 as an early PSII assembly factor
Source: Plant Cell. 2025 Mar 17;37(6):koaf055. doi: 10.1093/plcell/koaf055 (PMC12164590; doi:10.1093/plcell/koaf055)
Supplement: koaf055_Supplementary_Data [file koaf055_supplementary_data.zip › psb28_interactive.zip!/plots/intensity/qualityPlots/peptides/CAG2_Cre06.g293850.t1.2.html]

### Cre06.g293850.t1.2

Trivial name: CAG2  
SeqCoverage [median]: 40.10  
Mass: 31.17 kDa  
  
 

| Sequence | MissedCleavage | Mass [Da] | wtIntensity | muIntensity | Score |
| --- | --- | --- | --- | --- | --- |
| AVPTGLNAATLIGEK | 0 | 1453.81 | 40091.14 | 52049.29 | 146.77 |
| AVVHAAR | 0 | 722.42 | 970.96 | 6629.18 | 130.84 |
| EALEPGTAWR | 0 | 1128.56 | 5472.62 | 4221.90 | 175.44 |
| IPSGELWGGSPAK | 0 | 1297.67 | 0.00 | 571.64 | 80.92 |
| LQHEAEALEK | 0 | 1166.59 | 35444.86 | 23587.43 | 161.94 |
| LQHEAEALEKLTH | 1 | 1517.78 | 1786.38 | 7793.55 | 88.55 |
| LVDQGEYEWINFR | 0 | 1667.79 | 3041.82 | 0.00 | 138.45 |
| QVLIEDEWYNR | 0 | 1463.70 | 7510.30 | 6481.81 | 134.25 |
| QVLIEDEWYNRQR | 1 | 1747.86 | 0.00 | 795.67 | 37.43 |
| RIPSGELWGGSPAK | 1 | 1453.77 | 2237.24 | 5303.52 | 66.00 |
| VLDDVSTHYHNLATMFR | 0 | 2017.97 | 12702.24 | 7579.46 | 92.58 |
| VTVEPYAVLR | 0 | 1145.64 | 29239.52 | 52930.57 | 175.38 |
|  |  |  | 138497.1 | 167944.0 |  |
